# A Novel Homozygous Frameshift Variant in *XYLT2* Causes Spondyloocular Syndrome in a Consanguineous Pakistani Family

**DOI:** 10.3389/fgene.2019.00144

**Published:** 2019-03-05

**Authors:** Mehran Kausar, Elaine Guo Yan Chew, Hazrat Ullah, Mariam Anees, Chiea Chuen Khor, Jia Nee Foo, Outi Makitie, Saima Siddiqi

**Affiliations:** ^1^Department of Biochemistry, Quaid-i-Azam University, Islamabad, Pakistan; ^2^Institute of Biomedical and Genetic Engineering (IBGE), Islamabad, Pakistan; ^3^Folkhälsan Institute of Genetics, Helsinki, Finland; ^4^Lee Kong Chian School of Medicine, Nanyang Technological University, Singapore, Singapore; ^5^Human Genetics, Genome Institute of Singapore, A^∗^STAR, Singapore, Singapore; ^6^National Institute of Rehabilitation Medicine (NIRM), Islamabad, Pakistan; ^7^Children’s Hospital, University of Helsinki and Helsinki University Hospital, Helsinki, Finland

**Keywords:** spondyloocular syndrome (SOS), whole-exome-sequencing (WES), osteoporosis, xylosyltransferase II (*XYLT2*), cataract

## Abstract

We report on three new patients with spondyloocular syndrome (SOS) in a consanguineous Pakistani family. All three patients present progressive generalized osteoporosis, short stature, recurrent fractures, hearing loss and visual impairments. WES revealed a novel homozygous frameshift variant in exon 11 of *XYLT2* (NG 012175.1, NP_071450.2) resulting in loss of evolutionary conserved amino acid sequences (840 – 865/865) at C-terminus p.R840fs^∗^115. Sanger Sequencing confirmed the presence of the novel homozygous mutation in all three patients while the parents were heterozygous carriers of the mutation, in accordance with an autosomal recessive inheritance pattern. Only nine variants worldwide have previously been reported in *XYLT2* in patients with SOS phenotype. These three patients with novel homozygous variant extend the genotypic and phenotypic spectrum of SOS.

## Introduction

Spondyloocular syndrome (OMIM 605822), a very rare form of autosomal recessive genetic skeletal dysplasia, was first defined and reported by Schmidt et al. (2001), Taylan et al. (2017). In recent years, spondyloocular syndrome (SOS) has been linked to variant in the xylosyltransferase II encoded by *XYLT2* (MIM 608125) (Munns et al., 2015; Taylan et al., 2016). XYLT2 is involved in the biosynthesis of proteoglycans (PGs).

PGs, surface-associated and extracellular matrix proteins, play a vital role in sustaining the homeostasis in various tissues including skin, bone and cartilage (Couchman and Pataki, 2012). PGs are composed of core protein on which glycosaminoglycan (GAGS) chains, made up of tetrasaccharide linkers, are assembled. Mainly two types of sulfated PGs, heparin sulfate PGs and chondroitin sulfate/dermatan sulfate PGs are involved (Couchman and Pataki, 2012). Synthesis of tetrasaccharide linker molecules is catalyzed by four consecutive enzymatic reactions, first reaction requiring the transfer of xylose from uridine diphosphate-xylose (UDP-Xyl) to particular serine residue of the core protein by xylosyltransferase I (MIM 608124) and II. Next step involves the addition of two galactose residues by galactosyltransferase I and II, encoded by *B4GALT7* (MIM 604327) and *B3GALT6* (MIM 615291), respectively. Final reaction involves the transfer of glucuronic acid by glucuronosyltransferase I, encoded by *B3GAT3* (MIM 606374). Variants in genes encoding these five enzymes have been reported in various genetic skeletal diseases, which are collectively known as linkeropathies (Mizumoto et al., 2015; Taylan et al., 2016).

To date, only nine variants in *XYLT2* have been identified in the patients with SOS phenotype worldwide (Taylan et al., 2017; Umair et al., 2017). Here we present a consanguineous Pakistani family with three children with moderate to severe manifestations of SOS due to a novel homozygous frameshift variant in *XYLT2*.

## Case Presentation and Method

**FIGURE 1:**
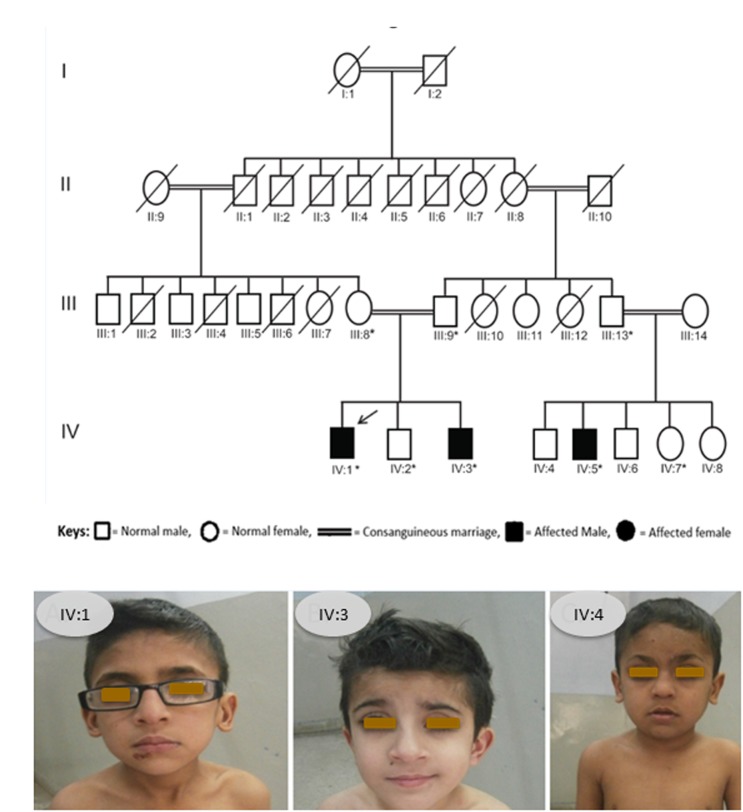
**(A)** Pedigree of affected family. Arrow indicates the affected member whose DNA sample was processed for WES and asterisks indicate the family members whose blood samples were collected and Sanger sequenced. **(B)** Photograph showing all the three patients from the SOS family IV:1, IV:3, and IV:4.

We identified a consanguineous Pakistani family in which three family members, 12 and 9 years old brothers (IV:1, IV:3) and their 10 years old cousin (IV:5), were affected with a rare skeletal dysplasia (Figure 1A,B). A written informed consent was obtained from the head of family (father) before the collection of blood samples for genetic analyses from all available family members and publication of the results.

All three patients were under treatment in Children’s Hospital Lahore, Pakistan. Growth parameters including height and weight of patients, were measured and compared with reference values (Aziz et al., 2012) among Pakistani population (Aziz et al., 2012). The clinical data, including radiographs, were assessed retrospectively. Genetic studies were performed using peripheral blood genomic DNA. We used 1μg of genomic DNA for targeted enrichment from Patient 1 (IV:1) using the Nimblegen SeqCap EZ Exome v3 kit and barcoded for sequencing on a single lane of a multiplexed 2x151 bp sequencing run on the Illumina HiSeq 2000 platform. This individual’s sample was sequenced to a mean coverage of 37.8 reads per target base, with 97% of the target exome covered by 10 or more reads. Reads were mapped using BWA v0.7.17 (Genomes Project et al., 2012) and variants were called using the GATK v2 Unified Genotyper following the recommended guidelines by GATK ‘Best practices for variant calling v3’ (Genomes Project et al., 2012). Variants present at ≤ 1% frequency in 1000 Genomes, HapMap and ExAC populations were identified from exome sequencing data. SIFT (Sim et al., 2012) and Polyphen2 (Adzhubei et al., 2010) were used to predict effect of each missense variant. The variant of interest was looked up within in-house exome data of 95 Pakistani individuals (16 normal individuals and 79 individuals with an unrelated disease phenotype) which were processed as described above. We used the following primer sequences for the segregation analysis of detected mutations in *XYLT2* by polymerase chain reaction and Sanger sequencing: *XYLT2*-Forward, GCAAGCTGTGACTCAGAAGTA and *XYLT2*-Reverse, AGCCTCGTGCAGAACAATAG.

## Results

### Clinical Findings

There were three patients in the family, the index Patient 1 (IV:1) is a 12 years old boy born to consanguineous healthy parents from uncomplicated pregnancy at full term. He presented with delayed milestones and multiple compression fractures at the age of 9 months. His first right femoral fracture occurred at 12 months and generalized osteoporosis was noted. Presently, his height is 124 cm (Z-score -2.4) and weight 36 kg (Z-score -0.2). Physical evaluation revealed a low posterior hairline, short and webbed neck, low set ears, shield chest, long fingers and toes. Sclerae were normal and no dental problems were observed. Radiographs revealed generalized osteoporosis, mild to moderate thoracic kyphosis, platyspondyly and increased intervertebral disk space. Intravenous pamidronate treatment was started at the age of 36 months and resulted in reshaping of some vertebrae. At the age of 12 years the patient is ambulant but has unsteady gait due to muscle weakness.

In addition to skeletal problems, the patient was diagnosed with moderate hearing loss at 8 years of age. Eye examination revealed nystagmus and amblyopia, and spontaneous left retinal detachment occurred. Cataracts were noted at 12 years. Learning difficulties were also obvious since early childhood. Biochemical analysis of patient’s blood revealed normal levels of calcium, alkaline phosphatase, creatinine and 25-OH-vitamin D.

The 2^nd^ patient (IV:3) is 9 years old, younger brother of patient 1, and had a similar clinical course and manifestations as his elder brother. He was diagnosed with generalized vertebral flattening and multiple compression fractures at the age of 6 months. Currently, his height is 109 cm (Z-score -3.5) and weight 29 kg (Z-score -0.7). He also had pamidronate infusions at the age of 15 months. Reshaping of some vertebrae were noted, but new compression fractures have also occurred. He has sustained recurrent long bone fractures but is ambulant. Mild to moderate hearing loss and vision impairment appeared at the age of 6 years and he also had learning difficulties. Biochemical analysis of patient’s blood revealed normal levels of calcium, alkaline phosphatase, creatinine and 25-OH-vitamin D.

The 3^rd^ patient of the family (IV:4) is a 10 years old boy who has had similar clinical course and manifestations as his two older cousins. Presently, his height is 111 cm (Z-score -3.7) and weight 33 kg (Z-score -0.4). Delayed milestones and multiple compression fractures were apparent at the age of 9 months. His first femoral fracture occurred at the age of 18 months and generalized osteopenia was observed; multiple other fractures have occurred thereafter. Physical evaluation revealed a low posterior hairline, short and webbed neck, low set ears, shield chest, long fingers and toes. Sclerae and teeth were normal. Radiographs revealed moderate thoracic kyphosis and platyspondyly. He started intravenous pamidronate treatment at the age of 3 years which improved the compression fractures but did not completely prevent new fractures. Along with skeletal problems he was also diagnosed with hearing loss and visual impairment at the age of 5 years. He was operated on for bilateral cataract. Learning difficulties were observed since early childhood. Blood biochemistry for calcium, alkaline phosphatase, creatinine and 25-OH-vitamin D was normal.

### Genetic Findings

**FIGURE 2:**
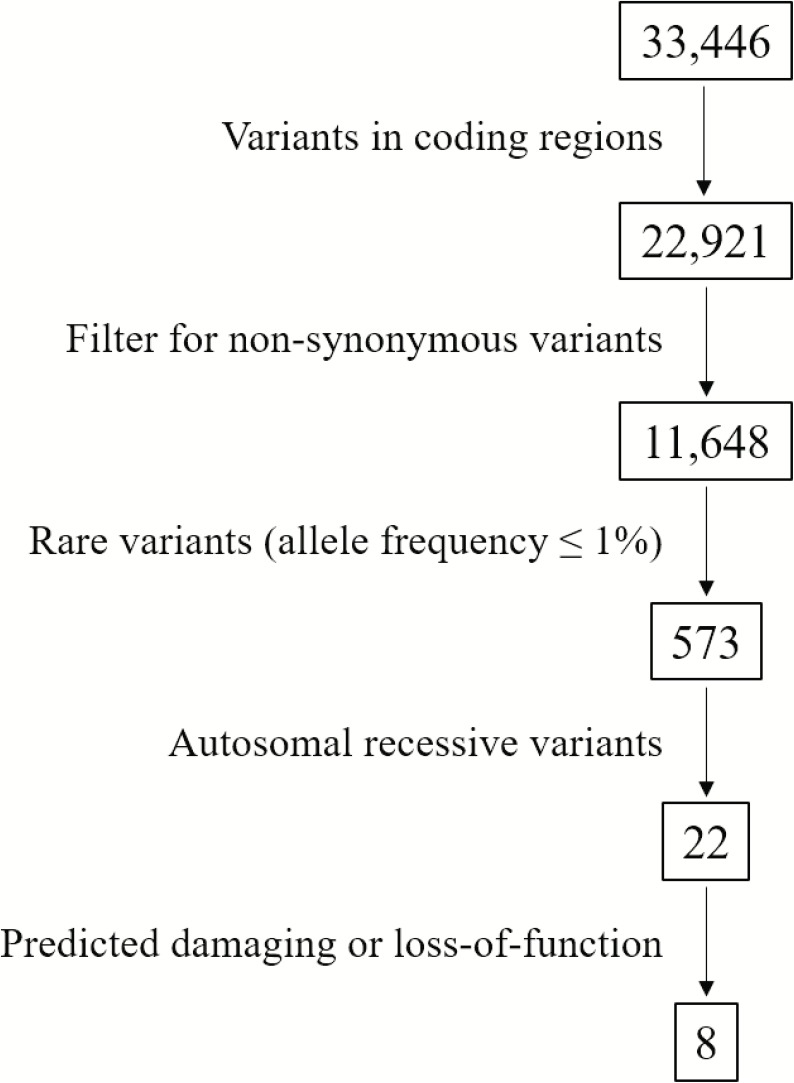
Flowchart of variant filtering. A total of 33,446 variants were found from WES of Patient 1. Eight candidate variants remain after filtering (Supplementary Table S1).

**FIGURE 3:**
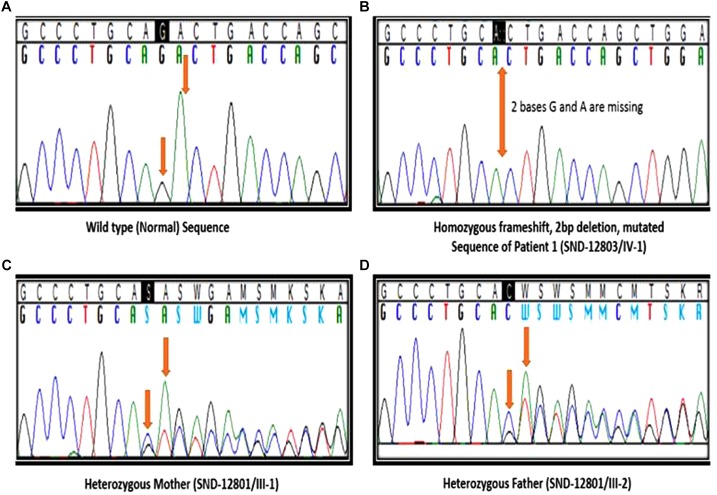
Segregation analysis. **(A)** Sanger sequencing results of healthy sibling (IV:2), arrows indicating no change. **(B)** Sequencing results of patient 1 (IV:1), arrow indicating homozygous deletion of 2 bases. **(C,D)** Sequencing results of father (III:8) and mother (III:9), arrows indicating heterozygous deletion.

We identified a total of 33,446 variants from WES of Patient #1. Eight variants remained after filtering for variants which localized to coding regions, are non-synonymous, rare (allele frequency ≤ 1%), autosomal recessive and predicted to be damaging (Figure 2, Supplementary Table S1). Based on the functions of the genes in which these 8 candidate variants localized and the previously reported gene association with SOS, we focused on the 17:g.48437571CAG>C deletion variant in *XYLT2* (xylosyltransferase II). The deletion g.48437572delAG (**p.R840Tfs^∗^115)** which is present at 17q21.33 alters the reading frame, substituting the last 25 amino acids (p.840 – 865) at carboxy terminal end of the xylosyltransferase II. Sanger sequencing confirmed that all three patients were homozygous for this variant and their parents were heterozygous (Figure 3). Two healthy siblings were negative for the variant. This variant was not present in exome-sequencing data of 95 Pakistani individuals, and was also not found in the available population databases, including the exome aggregation consortium database (ExAC) and genome aggregation database (gnomAD). This novel variant segregated perfectly with SOS manifestations and was regarded as the cause of the patient’s phenotype.

## Discussion

We report the phenotypic characteristics and molecular diagnosis of SOS in three children from a consanguineous Pakistani family. All three patients present with generalized osteoporosis, multiple compression fractures, hearing loss and visual impairment, all consistent with the diagnosis of SOS. We report a novel homozygous pathogenic frameshift variant in *XYLT2* in the family under study. Chromosomal location of the *XYLT2* is 17q21.3-q22. *XYLT2* contains 11 exons and 10 introns, and encodes an 865 amino acids long protein. The XYLT2 is comprised of four domains; N-terminus domain, catalytic domain, a core2/I-branching enzyme domain and a C-terminus domain (Umair et al., 2017). Schmidt et al. (2001) published the first clinical report on SOS in a consanguineous Iraqi family with 6 affected individuals. Alanay et al. (2006) reported in 2006 another patient with SOS in a Turkish family. Munns et al. (2015) identified a pathogenic biallelic frameshift insertion in *XYLT2* as the genetic cause of SOS in a non-consanguineous Australian European family with two affected brothers; another patient had a deleterious homozygous frameshift deletion. Taylan et al. (2016) identified a nonsense pathogenic frameshift variant (p.Arg730^∗^) in a Turkish patient and two different pathogenic missense variants (p.Arg563Gly and p.Leu605Pro) in consanguineous Canadian and Iraqi families. To date, two homozygous frameshift deletions, one homozygous frameshift insertion, one homozygous nonsense and five homozygous missense variants in *XYLT2* with typical SOS phenotypic characteristics have been reported (Taylan and Makitie, 2016; Taylan et al., 2017; Umair et al., 2017; Table 1). Here, we report a novel homozygous nonsense frameshift variant in a consanguineous Pakistani family with three affected individuals with manifestations of SOS including moderate to severe early-childhood onset osteoporosis, multiple compression fractures, gradual hearing loss and visual impairment. Our patients had a frameshift change in exon 11 of *XYLT2*. The p.Ala174Profs^∗^35 and p.Val232Glyfs^∗^54 frameshifts previously reported by Munns et al. (2015) in exon2 and 3 lead to premature termination and truncated mRNA (Table 1). None of our patients currently present with severe cardiac problems or congenital heart defects. The described frameshift **p.R840Tfs^∗^115,** is predicted to adversely affect the catalytic subunit of xylosyltransferase II. The described frameshift results in the removal of the last 25 evolutionarily conserved amino acids in the last exon of *XYLT2* (exon11). Defects in exon 11 inhibit the catalysis of sugar transfer to serine residue of PG core proteins thus resulting in improper PGs synthesis. Phenotypic features in our patients are more serious as compared with those reported in subjects with homozygous missense mutations by Taylan et al. (2016), It is possible that the single amino acid replacement described by Taylan et al. (2016) may allow residual protein function while in our patients the removal of 25 amino acids may result in complete loss of XYLT2 function. All these patients have been treated with pamidronate infusions, which has helped in reducing the fractures but some new compression fractures still occurred. As expected, pamidronate infusion did not improve vision or hearing.

**Table 1:** All reported cases of SOS with *XYLT2* mutations and phenotypic differences.

	This report			Umair et al., 2017		Taylan et al., 2017		Taylan et al., 2016				Munns et al., 2015		
	Patient1	Patient2	Patient3	Family A	Family B	Patient1	Patient2	Patient1	Patient2	Patient3	Patient 4	Patient1	Patient2	Patient3
Ethnicity	Pakistani	Pakistani	Pakistani	Iraqi	Turkish	Turkish	Turkish	Turkish	Canadian		Iraqi	European Australian		
Consanguinity	+	+	+	+	-	-	+	+	+	+	+	-	-	+
Mutation	c.2518 – 2519delAG p.R840fs^∗^115			c.1159C>T p.R387W	c.2548G>C p.D850H	p.M237R	p.E854Afs^∗^101	c.2188C>T p.R730^∗^	c.1687C>Gp.R563G	c.1814T>C p.L605P	c.692dupC p.V232Gfs^∗^54	c.692dupC p.V232Gfs^∗^54	c.520delA p.A174Pfs^∗^35
Skeletal fragility	+	+	+	+	+	+	+	+	+	+	+	+	+	+
Recurrent fractures	+	+	+	+	+	+	+	+	+	+	+	+	+	+
Decreased spine mobility	+	+	+	+	+	+	+	+	+	+	+	+	+	+
Facial dysmorphism	+	+	+	+	+	+	+	+	+	+	+	+	+	+
Platyspondyly	+	+	+	+	+	+	+	+	+	+	+	+	+	+
Cataract	+	+	+	+	+	+	+	+	+	+	+	+	+	+
Ureter dilation	-	-	-	-	-	-	-	-	-	-	-	+	+	-
Hearing loss	+	+	+	-	-	+	+	-	-	-	-	+	+	+
Learning difficulties	+	+	+	NA	+	+	+	+	+	+	+	+	+	-
Dental problems	-	-	-	-	-	-	-	-	+	-	-	-	-	-
Cardiac issues	-	-	-	+/-	-	-	-	+	-	-	-	+	+	-
Effect of bisphosphonate therapy	+/-	+/-	+/-	NA	NA	+	+	+	+	+	+	+	+	+/-


To date, all patients including our patients with SOS manifestation and defect in xylosyltransferase II have showed autosomal recessive pattern of inheritance, and no clinical manifestations have been reported in a heterozygous state. This finding suggests that only biallelic deleterious variants in *XYLT2* lead to such a loss in enzyme function that clinical symptoms appear, while in a heterozygous state the XYLT2 function is compensated by the normal allele.

## Conclusion

We identified a novel deleterious nonsense frame shift variant in the *XYLT2* in three children in a consanguineous Pakistani family. This novel variant results in marked skeletal and extra-skeletal features including generalized osteoporosis, platyspondyly, gradual hearing loss, and visual impairment. Differences in phenotypic presentations, from mild to severe forms, between our and previously reported patients are likely to be dependent on the nature and location of the variant along with the expression pattern of *XYLT2* in different tissues. Pamidronate therapy was beneficial but did not fully restore the bone health.

## Data Availability

The raw data supporting the conclusions of this manuscript will be made available by the authors, without undue reservation, to any qualified researcher.

## Ethics Statement

Ethical committee from IBGE provided the ethical approval for our project on the Genetics of Rare Syndromes. Consent was also obtained from the head of the family for the publication of the results.

## Author Contributions

MK and SS did the family collection. HU and OM did the clinical evaluation. EC, CK, SS, and JF did the NGS data analysis. MK, EC, MA, JF, SS, and OM compiled the data and wrote the manuscript.

## Conflict of Interest Statement

The authors declare that the research was conducted in the absence of any commercial or financial relationships that could be construed as a potential conflict of interest.

## References

[B1] Adzhubei I. A. Schmidt S. Peshkin L. Ramensky V. E. Gerasimova A. Bork P. (2010). A method and server for predicting damaging missense mutations. *Nat. Methods* 7 248–249. 10.1038/nmeth0410-248 20354512 PMC2855889

[B2] Alanay Y. Superti-Furga A. Karel F. Tuncbilek E. (2006). Spondylo-ocular syndrome: a new entity involving the eye and spine. *Am. J. Med. Genet. A* 140 652–656. 10.1002/ajmg.a.31119 16470687

[B3] Aziz S. Noor-Ul-Ain W. Majeed R. Khan M. A. Qayum I. Ahmed I. (2012). Growth centile charts (anthropometric measurement) of Pakistani pediatric population. *J. Pak. Med. Assoc.* 62 367–377. 22755283

[B4] Couchman J. R. Pataki C. A. (2012). An introduction to proteoglycans and their localization. *J. Histochem. Cytochem.* 60 885–897. 10.1369/0022155412464638 23019015 PMC3527888

[B5] Genomes Project C. Abecasis G. R. Auton A. Brooks L. D. DePristo M. A. Durbin R. M. (2012). An integrated map of genetic variation from 1,092 human genomes. *Nature* 491 56–65. 10.1038/nature11632 23128226 PMC3498066

[B6] Mizumoto S. Yamada S. Sugahara K. (2015). Mutations in biosynthetic enzymes for the protein linker region of chondroitin/dermatan/heparan sulfate cause skeletal and skin dysplasias. *Biomed. Res. Int.* 2015:861752. 10.1155/2015/861752 26582078 PMC4637088

[B7] Munns C. F. Fahiminiya S. Poudel N. Munteanu M. C. Majewski J. Sillence D. O. (2015). Homozygosity for frameshift mutations in XYLT2 result in a spondylo-ocular syndrome with bone fragility, cataracts, and hearing defects. *Am. J. Hum. Genet.* 96 971–978. 10.1016/j.ajhg.2015.04.017 26027496 PMC4457947

[B8] Schmidt H. Rudolph G. Hergersberg M. Schneider K. Moradi S. Meitinger T. (2001). Retinal detachment and cataract, facial dysmorphism, generalized osteoporosis, immobile spine and platyspondyly in a consanguinous kindred–a possible new syndrome. *Clin. Genet.* 59 99–105. 10.1034/j.1399-0004.2001.590206.x 11260210

[B9] Sim N.-L. Kumar P. Hu J. Henikoff S. Schneider G. Ng P. C. (2012). SIFT web server: predicting effects of amino acid substitutions on proteins. *Nucleic Acids Res.* 40 W452–W457. 10.1093/nar/gks539 22689647 PMC3394338

[B10] Taylan F. Costantini A. Coles N. Pekkinen M. Heon E. Siklar Z. (2016). Spondyloocular syndrome: novel mutations in XYLT2 gene and expansion of the phenotypic spectrum. *J. Bone Miner. Res.* 31 1577–1585. 10.1002/jbmr.2834 26987875

[B11] Taylan F. Makitie O. (2016). Abnormal proteoglycan synthesis due to gene defects causes skeletal diseases with overlapping phenotypes. *Horm. Metab. Res.* 48 745–754. 10.1055/s-0042-118706 27871115

[B12] Taylan F. Yavas Abali Z. Jantti N. Gunes N. Darendeliler F. Bas F. (2017). Two novel mutations in XYLT2 cause spondyloocular syndrome. *Am. J. Med. Genet. A* 173 3195–3200. 10.1002/ajmg.a.38470 28884924

[B13] Umair M. Eckstein G. Rudolph G. Strom T. Graf E. Hendig D. (2017). Homozygous XYLT2 variants as a cause of spondyloocular syndrome. *Clin. Genet.* 93 913–918. 10.1111/cge.13179 29136277

